# Disparities in time to treatment initiation of invasive lung cancer among Black and White patients in Tennessee

**DOI:** 10.1371/journal.pone.0311186

**Published:** 2025-01-03

**Authors:** Lohuwa Mamudu, Saanie Sulley, Paul H. Atandoh, Joanne L. Reyes, Raquibul A. K. M. Bashar, Martin Whiteside, Archana J. McEligot, Hadii M. Mamudu, Faustine Williams

**Affiliations:** 1 Department of Public Health, California State University, Fullerton, Fullerton, CA, United States of America; 2 National Healthy Start Association, Washington, DC, United States of America; 3 Department of Mathematics, Mercer University, Macon, GA, United States of America; 4 Department of Mathematics and Computer Science, Augustana College, Rock Island, IL, United States of America; 5 Tennessee Department of Health, Tennessee Cancer Registry, Nashville, TN, United States of America; 6 Department of Health Services Management and Policy, College of Public Health, East Tennessee State University, Johnson City, TN, United States of America; 7 Center for Cardiovascular Risk Research, College of Public Health, East Tennessee State University, Johnson City, TN, United States of America; 8 Division of Intramural Research, National Institute on Minority Health and Health Disparities, National Institutes of Health, Bethesda, MD, United States of America; University of Saskatchewan, CANADA

## Abstract

**Background:**

Early initiation of treatment for lung cancer has been shown to improve patient survival. The present study investigates disparities in time to treatment initiation of invasive lung cancer within and between Black and White patients in Tennessee.

**Methods:**

A population-based registry data of 42,970 individuals (Black = 4,480 and White = 38,490) diagnosed with invasive lung cancer obtained from the Tennessee Cancer Registry, 2005–2015, was analyzed. We conducted bivariate ANOVA tests to examine the difference in time to treatment initiation among independent factors, and multivariable Cox proportional hazard models to identify independent factors that influence median time to treatment initiation after diagnosis.

**Results:**

When considering the estimate of the proportion of time to treatment initiation based on the combined influence of all independent factors (sex, age, race, marital, county of residence, health insurance, cancer stage, and surgical treatment), Black patients were generally more at risk of delayed treatment compared to Whites. Black patients aged <45 years (adjusted hazard ratio [aHR] = 1.40; 95% confidence interval [CI] = 1.01–1.94) and married White patients (aHR = 1.13; 95% CI = 1.07–1.18) had the highest increased risk of late treatment among their respective racial subgroups. In the general sample, patients with private health insurance had (aHR = 1.08; 95% CI = 1.01–1.16) higher risk of late treatment beyond 2.7 weeks compared to self-pay/uninsured patients. This was consistent among both Black and White subsamples. Patients with localized and regional lung cancer stages had a decreased risk of delayed treatment compared to those diagnosed at the distant stage among both Black and White patients.

**Conclusions:**

Black patients were often at greater risk of late initiation of treatment for invasive lung cancer in Tennessee. Additional research is needed to understand factors influencing time to treatment initiation for Black patients in Tennessee. Further, cancer care resources are needed in Black communities to ensure timely treatment of invasive lung cancer, reduce disparities, and promote equitable care for all cancer patients.

## Introduction

Lung cancer is the leading cause of cancer mortality in the United States (U.S.) [1]. With an estimated 154,050 deaths per year, lung cancer accounts for approximately 25% of all cancer deaths [2]. The prognosis for lung cancer differs significantly between cases depending on the progression of the disease and subtype. Lung cancer is categorized into two main types: non-small cell lung cancer and small cell lung cancer. Non-small cell lung cancer accounts for approximately 85% of lung cancer cases, with a reported 5-year survival rate of 26.5% [3]. Small cell lung cancer, the less common type, accounts for only 15% but is more aggressive and metastasizes rapidly, resulting in a lower 5-year survival rate of 6.7% [3].

Treatment of lung cancer varies from patient to patient depending on the stage at diagnosis, type, and progression [4]. Treatment methods such as surgical resection, chemotherapy, and radiation therapy are all known to improve survival rates among patients, with surgical treatment demonstrating the best outcomes [5–7]. Delays in treatment refer to the time elapsed between a diagnosis a start of definitive treatment. Several studies have shown that early detection and treatment of lung cancer offers 65–90% 5-year survival duration, especially at the localized stage (small tumor lung cancer) [8, 9]. Studies have shown that the delay in initiating lung cancer treatment ranges from 2–10 weeks [10–12], which can impact patient survival. However, there are conflicting findings about the effect of treatment delays on lung cancer patient survival. Prolonged delays have been linked to accelerated disease progression and poorer survival outcomes [13]. On the other hand, a retrospective study by Salomaa et al. (2005) found that a longer delay in a specialist treatment was associated with better survival in advanced-stage lung cancer [12]. Similarly, Anggondowati et al. (2020), used a national cancer database and found that a time to treatment of 4.1 to 6 weeks was associated with a lower risk of death for early-stage localized non-small cell lung cancer compared to treatment within 1 day to 4 weeks after diagnosis [14]. However, a subset analysis of their findings revealed that an extended time to surgery for early-stage disease was associated with a higher risk of death [14].

Despite the improving survival rates in lung cancer, health disparities in treatment persist. Using data from the National Center for Health Statistics, Yao et al. (2017) found that disparities continue in the U.S., with rural Appalachia region experiencing higher incidence and mortality rates of cancers than urban non-Appalachian region [15]. While the rate of lung cancer among Blacks is lower than among Whites in Tennessee (68 per 100,000 people vs 77 per 100,000), Black patients have a lower 5-year survival rate than Whites (19% vs. 22%) [16]. Recent studies in Tennessee and using national databases, respectively, have reported Black patients are significantly less likely to be recommended surgical treatment than White patients, resulting in lower survival rates than White individuals [16–19]. Beyond racial disparities, other sociodemographic factors substantially influence the time to treatment and prolonged delay [10, 11, 20–24]. In addition, higher socioeconomic status (SES) has been linked to better outcomes, while lower SES is associated with poorer outcomes [25]. Although previous research has examined the impact of sociodemographic factors on treatment delays and survival rates among lung cancer patients, there remains a notable gap in understanding these dynamics among Tennessean patients. This is particularly concerning, as Tennessee reports significantly higher rates of new lung cancer cases compared to the national average (73% vs. 57%) [26]. The lack of research focused on Tennessee is critical, given the state’s elevated lung cancer incidence and the potential for sociodemographic factors—such as race, income, and access to healthcare—to disproportionately affect treatment outcomes in this region. Expanding research efforts in Tennessee could provide valuable insights for addressing these disparities.

The present study is the first to investigate the influence of sociodemographic factors, type of insurance coverage, cancer stage, and surgical treatment on time to treatment initiation disparities (i.e., treatment time from diagnosis) of invasive lung cancer in Tennessee comparing outcomes within and between Black and White patients. We hypothesized that Black patients with invasive lung cancer would experience longer times to treatment initiation than their White counterparts. This study has the potential to enhance treatment strategies for lung cancer, leading to improved quality of life and survival outcomes for patients diagnosed with the disease.

## Materials and methods

### Study population and data

**Fig 1:**
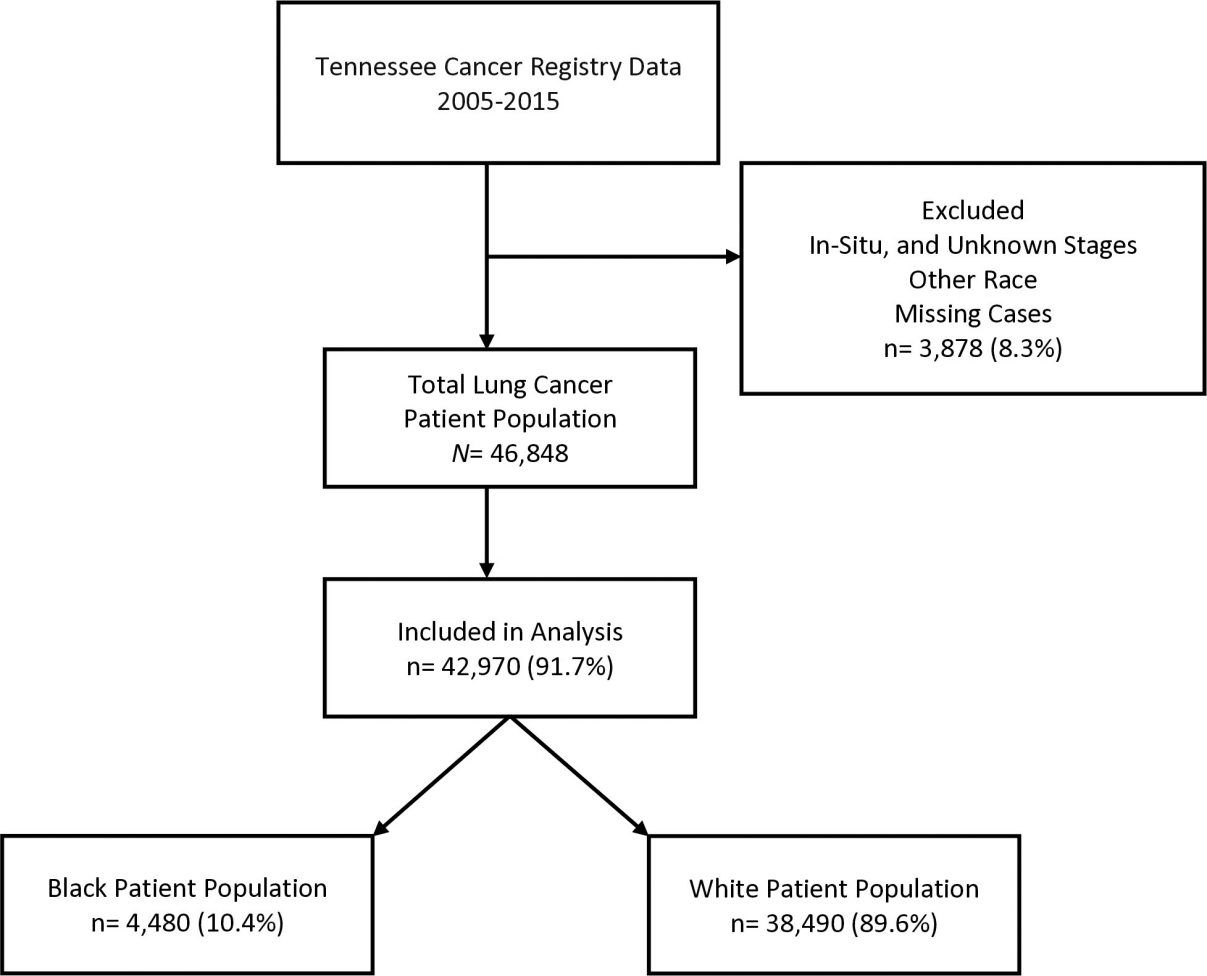
Flowchart illustrating criteria for patient inclusion and exclusion.

We obtained a retrospective population-based registry data of 46,848 Tennessee residents diagnosed with histologically confirmed invasive (malignant) or non-invasive lung cancer as the primary site of diagnosis as coded by the International Classification of Diseases for Oncology, Third Edition (ICD-O-3), from January 1, 2005, to December 31, 2015. The data was reported by the Tennessee Cancer Registry (TCR), which is a population-based, central cancer registry established and responsible for collecting and monitoring cancer incidence by Tennessee law [27]. We conducted a complete analysis of 42,970 cases of invasive lung cancer that received treatment or procedure within 12 months (52 weeks) and were diagnosed at the localized, regional, and distant stages, excluding the in-situ, and unknown stages of the lung cancer. The in-situ stage is considered pre-cancerous (i.e., not a true cancer) and non-invasive [28]. We also excluded other races and all cases with missing data for any of the selected variables from our analysis. Therefore, a total of 8.3% of the data (3,878 cases) were excluded. See Fig 1 for the sampling inclusion and exclusion procedure of patients. We focused on invasive lung cancer, because it tends to spread to other lymph nodes and, therefore, can significantly impact patient survival [3, 19], a crucial factor in lung cancer treatment. Data are available by request to the Tennessee Department of Health-TCR [29]. All analytical files are also available by reasonable request and Tennessee Department of Health-TCR approval.

### Measures

#### Dependent variable or outcome

The study’s dependent variable is the time to treatment initiation of invasive lung cancer, defined as the time from diagnosis to the start of definitive treatment. The time from diagnosis to treatment was measured as the median time to definitive treatment (in weeks); with a treatment delay defined as >2.7 weeks (i.e., treatment delay) [12, 30].

#### Independent variables

The independent variables included sociodemographic characteristics, race, age at diagnosis, marital status, county of residence, type of health insurance, stage of invasive lung cancer, and whether patients received surgical treatment. Age was categorized into 5 groups: <45; 45–54; 55–64, 65–74, and ≥75 years based on the aging criteria by the National Institute of Aging [31], and race as Black and White. Marital status was classified as single/never married, married/common-law, divorced/separated, and widowed. Type of insurance was grouped as public (Medicaid, Medicare, Indian Health Service, Veterans’ Affairs), private (fee for service, Health Maintenance Organization [HMO], Managed Care, and Preferred Provider Organization [PPO]), and self-pay/uninsured. The place/county of residence included whether an individual patient lived in an Appalachian (i.e., 52 counties) or non-Appalachian (i.e., 43 counties) region in Tennessee [32]. Stages of cancer included localized, regional, and distant stages. The Surgical procedures patients received included the following. Local tumor destruction, laser ablation or cryosurgery, laser excision, wedge resection, lobectomy with mediastinal lymph node dissection, lobe or bilobectomy with chest wall, extended pneumonectomy, extended pneumonectomy plus pleura or diaphragm, resection of lung, not otherwise specified (NOS), and surgery, NOS. Patients were recategorized as yes (having received surgery for the invasive lung cancer) or no surgery procedure.

### Ethical approval

The research protocol was approved by the Tennessee Department of Health Institutional Review Board on February 1st, 2018 (TDH-IRB 1057486), with continuation approval on June 15, 2023 (TDH-IRB 2020–0152). The National Institutes of Health–Intramural Research Program IRB–Human Research Protections Program–Office of Human Subjects Research Protections determined that the research protocol for this study did not involve human subjects, and thus was exempt from IRB review (18-NIMHD-00722). The anonymized data was received from TDH on March 21, 2018.

### Statistical analysis

**Fig 2:**
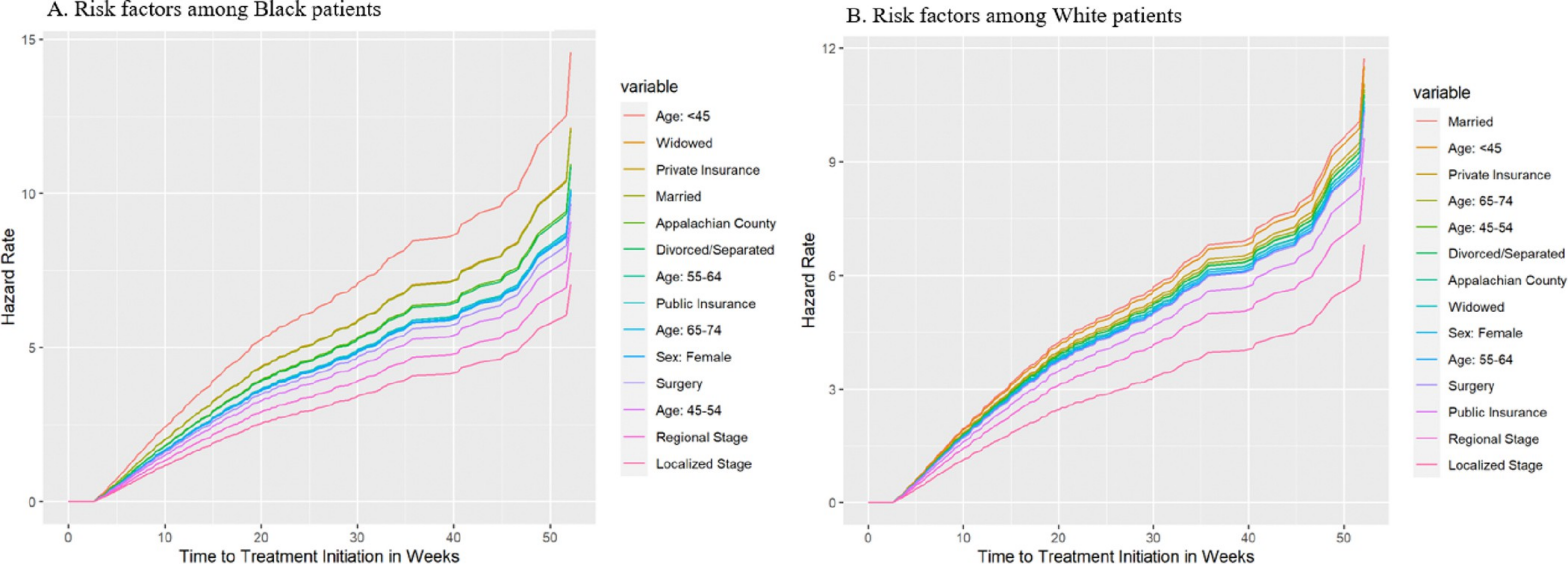
**A, B.** Ranking risk factors influencing time to treatment initiation of invasive lung cancer among patients in Tennessee (2005–2015).

**Fig 3:**
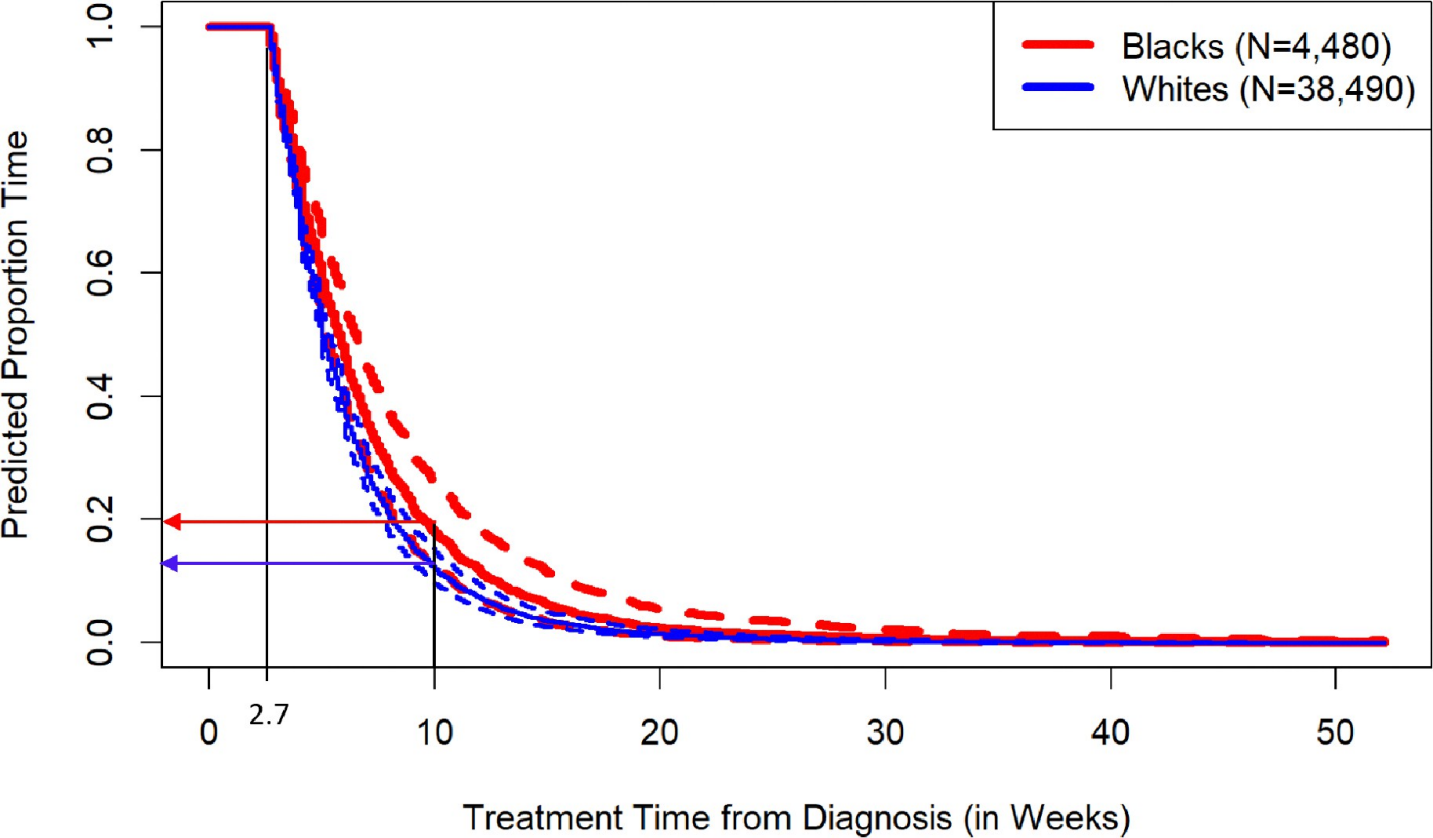
Predicted analysis of time-to-treatment initiation beyond 2.7 weeks from diagnosis among Black and White patients. Data from Tennessee Cancer Registry (2005–2015). Estimates were derived from an adjusted multivariable Cox Proportional Hazard model, incorporating the following risk factors: sociodemographic characteristics (sex, age, marital status, and county of residence), health insurance, stage of cancer, and surgical treatment. Thick line represents the predicted treatment time, and the dotted lines indicate the 95% confidence interval of the predicted treatment time. For instance, the predicted likelihood of receiving treatment 10 weeks after diagnosis was approximately 20% for Black patients and 17% for White patients.

We conducted frequencies to examine the sample descriptive characteristics of the independent variables. The median and interquartile range (IQR) were used to assess the distribution of age at diagnosis and time to treatment initiation (see Table 1). Next, we performed bivariate ANOVA tests to assess the variations of time to treatment initiation within factors (see Table 1). The ANOVA tests were conducted to examine the within-independent group variation of time to treatment initiation. The ANOVA test was validated using the test of homogeneity of variance based on the median and robust test, including the Welch and Brown-Forsythe test. Given the skewed data of time to treatment initiation, we repeated the ANOVA analysis using non-parametric Kruskal Wallis to test the difference or variation in time to treatment initiation within factors or independent variables (see S1 Table). Multivariable Cox-Proportional Hazards (Cox-PH) model analyses were conducted to examine the influence of the independent factors on time to treatment initiation. The Cox-PH model was conducted to examine the likelihood of invasive lung cancer patients delaying time to treatment initiation or receiving treatment beyond the median weeks after diagnosis in the combined or overall sample (see Table 2) and among stratified subgroups of Black and White patients (see Table 3). To conduct the Cox-PH regression model of the delayed time to treatment initiation, we used the time from diagnosis to definitive treatment. The event interest was defined as a delay in treatment initiation beyond the median time of 2.7 weeks. Time to treatment initiation was coded as “0” if treatment was initiated within ≤2.7 weeks and “1” if after >2.7 weeks. This allows us to estimate the hazard ratio to assess the prognosis, direction, and magnitude of association between the independent variables and delayed time to treatment initiation beyond 2.7 weeks. Additionally, we repeated the analyses of Tables 2 and 3 using the IQR weeks as the event in a supplemental analysis (see S2 and S3 Tables). We then validated the Cox-PH models by conducting and assessing the Cox-PH assumptions (see S1 Methods). A non-statistical significance (i.e., p>0.05) of independent variables (or covariates) and the GLOBAL test indicated the Cox-PH assumption was satisfied or valid. Additionally, Schoenfeld residual tests were visualized to assess influential observations and further validate the Cox-PH assumption. If the Schoenfeld residual test of independent variables shows most data are within a 95% confidence interval and non-statistical significance (i.e., p>0.05), it is also an indication of a satisfied or valid Cox-PH assumption. The final data analyzed was based on complete data for each of the included variables. The results from the statistical analysis are reported using the adjusted hazard ratio (aHR) with a 95% confidence interval (CI) and statistical significance at a p< α = 0.05 level of significance. We used figures to display hazard functions and further assess the association between independent factors and time to treatment initiation, ranking the HRs in descending order of the independent factors to determine the magnitude and extent of risk on time to treatment initiation within the subsample of Black and White patients (see Fig 2). We also compared the predicted estimated likelihood proportion of time to treatment initiation of invasive lung cancer beyond 2.7 weeks from the joint influence of all the independent variables among Black and White patients (see Fig 3). All analyses were conducted using IBM SPSS Statistics 28 Premium and R 4.0.2.

**Table 1:** Descriptives and bivariate ANOVA analysis of time to treatment initiation of invasive lung cancer; N = 42,970.

Characteristics	Total Patient Sample *N* = 42970)		White Patient (n = 38490)		Black Patients (n = 4480)	
	**Range; (IQR)**	**Median**	**Range; (IQR)**	**Median**	**Range; (IQR)**	**Median**
Age at diagnosis	1, 102; (60, 74)	67	1,100; (60, 75)	68	18, 102; (57, 72)	65
Time to treatment in weeks	0, 52.14; (0.7, 5.5)	2.71	0, 52.14; (0.7, 5.4)	2.71	0,52.14; (0.6, 6.1)	2.71
**Independent Variables**	**n (%)**	**ANOVA** **P-value**	**n (%)**	**ANOVA** **P-value**	**n (%)**	**ANOVA** **P-value**
**Sex**		0.417		0.650		**<0.001**
Male	24911 (55.6)		21411 (55.6)		2500 (55.8)	
Female	19095 (44.4)		17079 (44.4)		1980 (44.2)	
**Age at Diagnosis**		**<0.001**		**<0.001**		**0.005**
<45	790 (1.9)		694 (1.8)		96 (2.1)	
45–54	4723 (11.03)		4041 (10.5)		682 (15.2)	
55–64	11219 (26.1)		9759 (25.4)		1460 (32.0)	
65–74	15761 (36.7)		14327 (37.2)		1434 (32.0)	
≥75	10477 (24.4)		9669 (25.1)		808 (18.0)	
**Race**		**<0.001**				
White	38490 (89.6)		-		-	
Black	4480 (10.4)		-		-	
**Marital Status**		**0.020**		**<0.001**		0.509
Single/Never Married	5276 (12.1)		3846 (10.0)		1330 (29.7)	
Married/Common Law	23913 (55.7)		22294 (57.9)		1619 (36.1)	
Divorced/Separated	6278 (14.6)		5543 (14.4)		735 (16.4)	
Widowed	7603 (17.7)		6807 (17.7)		796 (17.8)	
**County of Residence**		**0.003**		**<0.001**		0.334
Appalachian	23470 (54.6)		22540 (58.6)		930 (20.8)	
non-Appalachian	19500 (45.4)		15,950 (41.4)		3550 (79.2)	
**Health Insurance Type**		**<0.001**		**<0.001**		0.525
Self-Pay/Uninsured	2113 (4.9)		1736 (4.5)		377 (8.4)	
Public	30230 (70.4)		27248 (70.8)		2982 (66.6)	
Private	10627 (24.7)		9506 (24.7)		1121 (25.0)	
**Cancer Stage**		**<0.001**		**<0.001**		**<0.001**
Local	9679 (22.5)		8832 (22.9)		847 (18.9)	
Regional	11907 (27.7)		10695 (27.8)		1212 (24.1)	
Distant	21384 (49.8)		18963 (49.3)		2421 (54.0)	
**Surgical Treatment**		**<0.001**		**<0.001**		**<0.001**
Yes	12023 (28.0)		10885 (28.3)		1138 (25.4)	
No	30947 (80.0)		27605 (71.7)		3342 (74.6)	
**Time to Treatment Initiation**		-		-		-
≤2.7 weeks	20913 (48.7)		18741 (48.7)		2172 (48.5)	
>2.7 weeks	22057 (51.3)		19749 (51.3)		2308 (51.5)	

Sample descriptives = Frequencies

Statistical analysis performed = ANOVA Tests

Public insurance = (Indian Health Service, Medicaid, Medicare, Veterans’ Affairs)

Private insurance = (Fee for Service, HMO, Managed Care, PPO)

Bold = Statistical significance, p <0.05.

**Table 2:** Multivariable Cox-proportional hazard model of time to treatment initiation of invasive lung cancer beyond 2.7 median weeks in the overall patient sample. *N* = 42,970.

Independent Description	aHR (95% CI)	P-value
**Sex**		
Male	Ref	-
Female	1.00 (0.97, 1.03)	0.975
**Age at Diagnosis**		**<0.001**
<45	1.14 (1.02, 1.27)	**0.023**
45–54	1.01 (0.96, 1.07)	0.622
55–64	1.00 (0.96, 1.04)	0.871
65–74	1.04 (1.01, 1.07	**0.026**
≥75	Ref	-
**Race**		**<0.001**
White	Ref	-
Black	0.82 (0.78, 0.85)	**<0.001**
**Marital status**		**<0.001**
Single/Never Married	Ref	-
Married/Common Law	1.13 (1.08, 1.19)	**<0.001**
Divorced/Separated	1.04 (0.99, 1.09)	0.140
Widowed	1.04 (0.98, 1.09)	0.196
**County of Residence**		
Appalachian	1.02 (0.99, 1.05)	0.163
non-Appalachian	Ref	-
**Health Insurance Type**		**<0.001**
Self-Pay/Uninsured	Ref	-
Public	0.94 (0.88, 1.01)	0.089
Private	1.08 (1.01, 1.16)	**0.034**
**Cancer Stage**		**<0.001**
Localized	0.66 (0.64, 0.68)	**<0.001**
Regional	0.82 (0.80, 0.85)	**<0.001**
Distant	Ref	-
**Surgical Treatment**		
Yes	0.99 (0.95, 1.02)	0.364
No	Ref	-

Statistical analysis performed = Multivariable Cox proportional hazard regression analysis.

Ref = Reference group; aHR = Adjusted Hazard ratio; CI = Confidence interval.

Public insurance = (Indian Health Service, Medicaid, Medicare, Veterans’ Affairs)

Private insurance = (Fee for Services, HMO, Managed Care, PPO).

Bold = Statistical significance, p <0.05.

**Table 3:** Multivariable Cox-proportional hazard model assessment of time to treatment initiation of invasive lung cancer beyond 2.7 median weeks among White and Black patients in Tennessee.

	White Patient Model (*n* = 38,490)		Black Patient Model *(n* = 4,480)	
Variable Description	aHR (95% CI)	P-value	aHR (95% CI)	P-value
**Sex**				
Male	Ref	-	Ref	-
Female	1.01 (0.97, 1.03)	0.705	0.958 (0.87, 1.04)	0.340
**Age at Diagnosis**		**0.021**		**0.049**
<45	1.11 (0.98, 1.24)	0.099	1.40 (1.00, 1.94)	**0.044**
45–54	1.04 (0.97, 1.09)	0.250	0.87 (0.73, 1.03)	0.109
55–64	1.00 (0.95, 1.04)	0.858	0.97 (0.84, 1.12)	0.721
65–74	1.05 (1.00, 1.08)	**0.013**	0.97 (1.84, 1.09)	0.593
≥75	Ref	-	Ref	-
**Marital Status**		**<0.001**		**0.023**
Single/Never Married	Ref	-	Ref	-
Married/Common Law	1.13 (1.07, 1.18)	**<0.001**	1.16 (1.04, 1.28)	**0.005**
Divorced/Separated	1.03 (0.97, 1.09)	0.268	1.04 (0.91, 1.18)	0.530
Widowed	1.02 (0.95, 1.07)	0.597	1.17 (1.01, 1.33)	**0.028**
**County of Residence**				
Appalachian	1.02 (0.99, 1.05)	0.211	1.05 (0.94, 1.16)	0.334
non-Appalachian	Ref	-	Ref	-
**Health Insurance Type**		**<0.001**		**0.006**
Self-Pay/Uninsured	Ref	-	Ref	-
Public	0.92 (0.85, 1.00)	0.053	0.97 (0.82, 1.14)	0.750
Private	1.06 (0.98, 1.14)	0.135	1.16 (0.98, 1.37)	0.076
**Cancer Stage**		**<0.001**		**<0.001**
Localized	0.65 (0.63, 0.68)	**<0.001**	0.68 (0.59, 0.76)	**<0.001**
Regional	0.83 (0.79, 0.85)	**<0.001**	0.78 (0.69, 0.86)	**<0.001**
Distant	Ref	-	Ref	-
**Surgical Treatment**				
Yes	0.99 (0.95, 1.02)	0.532	0.93 (0.83, 1.03)	0.164
No	Ref	-	Ref	-

Statistical analysis performed = Multivariable Cox proportional hazard regression analysis

Ref = Reference group; aHR = Adjusted Hazard ratio; CI = Confidence interval

Public insurance = (Indian Health Service, Medicaid, Medicare, Veterans’ Affairs)

Private insurance = (Fee for Service, HMO, Managed Care, PPO)

Bold = Statistical significance, p <0.05.

## Results

### Population characteristics and bivariate assessment of independent factors

In Table 1, we examined the population characteristics and statistical variation in time to treatment initiation within independent groups. Among the total patients (*N =* 42,970) diagnosed with invasive lung cancer, 48.7% initiated treatment within 2.7 median weeks, while 51.3% started treatment after 2.7 weeks. In the subgroup analysis, nearly equal percentages of White (48.7%) and Black (48.5%) patients began treatment within 2.7 median weeks of diagnosis. A similar pattern was observed among those who initiated treatment after the 2.7 median weeks, with 51.3% being White and 51.5% being Black patients. The median age at diagnosis for the total sample was 67 years old (Whites = 68; Blacks = 65). The sample consisted of 24,911 (55.6%) males (Whites = 55.6%; Blacks = 55.8%) and 19,095 (44.4%) females (Whites = 44.4%; Blacks = 44.2%). Majority of the patients did not receive surgical treatment (72.0%; n = 30,947 [Whites = 71.7%; Blacks = 74.6%]), mostly married/common law (55.7%; n = 23,913 [Whites = 57.9%; Blacks = 36.1%]), resided in Appalachian county (54.6%; n = 23,470 [Whites = 58.6%]), enrolled in public insurance (70.4%; n = 30,230 [Whites = 70.8%; Blacks = 66.6%]), and were diagnosed with invasive lung cancer at the distant stage (49.80%; n = 21,384 [Whites = 49.3%]). Additionally, most Black patients lived in non-Appalachian county (79.3%) and were diagnosed with regional-stage lung cancer (54.0%). In the overall sample, the bivariate analysis showed a statistically significant difference in time to treatment initiation with age (p<0.001), race (p<0.001), marital status (p = 0.020), county of residence (p = 0.003), health insurance type (p<0.001), surgical treatment (p<0.001), and cancer stage (p<0.001), which is consistent with the White patients subgroup. Among Black patients, a statistically significant differences in time to treatment initiation were observed based on sex (p<0.001), age (p = 0.005), cancer stage (p<0.001), and surgical treatment (p<0.001), but not for marital status, county of residence, and health insurance type (see Table 1). The analysis in Table 1 was repeated using a non-parametric Kruskal-Wallis test to examine the differences in the time to treatment initiation of invasive lung cancer across the levels of independent variables, revealing consistent results (see S1 Table).

### Multivariable analyses of independent factors and time to treatment initiation of invasive lung cancer in the general patient population

Table 2 assesses the influence of the independent factors on time to treatment initiation after 2.7 median weeks in the entire sample of invasive lung cancer patients in Tennessee. Patients aged <45 years were 14% more likely to start treatment after 2.7 weeks (aHR = 1.14; 95% CI = 1.02–1.27). Black patients were 18% less likely to experience delayed treatment (aHR = 0.82; 95% CI = 0.78–0.85) compared to White patients. Married patients had a 13% higher risk of beginning treatment after 2.7 weeks (aHR = 1.13; 95% CI = 1.08–1.19) compared to single patients. Patients with private insurance were 8% more likely to start treatment start after 2.7 weeks (aHR = 1.08; 95% CI = 1.01–1.16) than those who were self-pay/uninsured. Individuals diagnosed with lung cancer at localized (aHR = 0.66; 95% CI = 0.64–0.68) and regional (aHR = 0.82; 95% CI = 0.80–0.85) stages were less likely to start treatment late compared to those diagnosed at the distant stage. In S2 Table, we repeated the Table 2 analysis to examine the influence of the independent factors on time to treatment initiation beyond 4.8 IQR weeks. Overall, we observed similar trends and statistical significance for most factors.

### Independent factors influencing time to treatment initiation of invasive lung cancer among White and Black patients

Table 3 examines the disparities and the influence of independent factors on the time to treatment initiation of invasive lung cancer among Black and White patients. Black married patients (aHR = 1.16; 95% CI = 1.04–1.28) and those aged <45 years (aHR = 1.40; 95% CI = 1.00–1.94) were more likely to start treatment after 2.7 weeks. Similarly, White married individuals (aHR = 1.13; 95% CI = 1.07–1.18), those aged <45 (aHR = 1.11; 95% CI = 0.98–1.24), and those aged 65–74 (aHR = 1.05; 95% CI = 1.00–1.08) were at an increased risk of late treatment beyond 2.7 weeks. There was an increased risk of starting treatment late for White (aHR = 1.02; 95% CI = 0.99–1.05) and Black (aHR = 1.05; 95% CI = 0.94–1.16) patients residing in Appalachian counties; however, this did not have a statistically significant influence on time to treatment initiation after 2.7 weeks.

Among both Black and White patients with private health insurance coverage, there was a higher risk of late treatment initiation; however, these findings ere not statistically significant. Notably, there was a significantly decreased risk of delayed treatment initiation for localized stage cancer among both Black patients (aHR = 0.68; 95% CI = 0.59–0.76) and White patients (aHR = 0.65; 95% CI = 0.63–0.68). For regional stage lung cancer, both Black (aHR = 0.78; 95% CI = 0.69–0.86) and White patients (aHR = 0.83; 95% CI = 0.79–0.85) were at a reduced risk of late treatment after 2.7 weeks. Surgical treatment had no statistically significant influence on time to treatment initiation after 2.7 weeks. However, Black patients who underwent surgical procedures had a greater decreased risk of late treatment compared to White patients (7% vs. 1%, respectively). In S3 Table, we repeated the Table 3 analysis to examine the disparities and influence of the independent factors on time to treatment initiation beyond the IQR weeks among White patients (4.7 weeks) and Black patients (5.5 weeks). We observed a similar magnitude, direction, and statistical significance in the associations for most independent factors, especially among White patients.

### Ranking of independent factors influencing time to treatment initiation of invasive lung cancer among Black and White patients

Fig 2 ranks the independent factors in order of their impact on time to treatment initiation beyond 2.7 weeks, from the most to the least impactful. Among Black patients, those aged <45 years were at the highest risk of delaying treatment after 2.7 weeks, followed by widowed individuals (see Fig 2A). Among White patients, married individuals were ranked as most at risk of delaying treatment, followed by those aged <45 years (see Fig 2B).

Both Black and White patients with private insurance were ranked third highest for delaying treatment in their populations. Appalachian Black and White patients were ranked fifth and seventh, respectively, while publicly insured Black and White patients were ranked eighth and twelfth, respectively, for delayed treatment.

Fig 3 displays the estimated predicted likelihood proportion of time to treatment initiation beyond 2.7 weeks after diagnosis among Black and White invasive lung cancer patients, respectively, as derived from the Cox-PHs models in Table 3. The hazard curve for Black patients mostly lies above that of White patients, considering all analyzed factors (sociodemographic, health insurance, stage of cancer, and surgical treatment) that influence the time to treatment initiation after 2.7 weeks. This implies that Black patients are generally at a higher risk of delaying treatment for invasive lung cancer (i.e., they had a higher predicted proportion time at a given treatment delay time beyond 2.7 weeks after diagnosis) than their White counterparts. For instance, the predicted likelihood of receiving treatment 10 weeks post-diagnosis is approximately 20% for Black patients and 17% for White patients.

## Discussion

The current study focuses on factors that influence the time to treatment initiation of lung cancer beyond 2.7 median weeks after diagnosis (i.e., treatment delay), considering the disparities between and within Black and White patients. We found that patients’ sociodemographic factors (age, race, marital status, and county of residence), health insurance status, and cancer stage (localized and regional) had a statistically significant influence on delayed time to treatment initiation after 2.7 weeks, while sex and surgical treatment did not. Our findings are consistent with past studies on lung cancer treatment delays [33, 34]. In the general Tennessee invasive lung cancer population, patients aged <45 years had a 14% increased risk, and those aged 65–74 years had a 4% increased risk of delayed treatment initiation beyond 2.7 weeks compared to those aged ≥75 years. However, in the stratified subgroup, only White patients aged 65–74 years showed a statistically significant increased risk, similar to what was observed in the general population sample.

Among Black patients, only those aged <45 years were statistically significantly associated with delayed treatment initiation beyond 2.7 weeks, showing a 40% increased risk compared to those aged ≥75 years. Additionally, compared to the general population, Black patients aged <45 years had a substantially higher risk difference of 26%, indicating that younger Black patients (<45 years) are at a greater risk of delaying treatment for invasive lung cancer. Contrary to our findings, a previous study by Samson et al. (2015) using the National Cancer Data Base reported that delayed treatment was associated with increasing age in their analysis of late surgery initiation and its impact on both short-term and long-term outcomes in early-stage non-small cell lung cancer [33]. The reasons behind the substantial delays in treatment among young Black patients in our study in Tennessee remain unclear. Interestingly, a multisite community intervention assessing cancer prevention educational messages in predominantly Black areas of Georgia (Atlanta & Decatur) and Tennessee (Chattanooga & Nashville) showed little or no effect on knowledge or attitudes in these intervention cities [35]. This adds to the uncertainty about why young Black patients face a higher risk of delaying treatment. Therefore, we recommend conducting an in-depth study to explore this issue further.

In the general (unstratified) population of Tennessee invasive lung cancer patients, Black patients were 18% less likely than White patients to delay treatment beyond 2.7 weeks. However, the stratified subpopulation analysis revealed that Black patients were at greater risk of late treatment compared to White patients across almost all independent factors considered. This finding is consistent with the predicted likelihood delayed treatment initiation beyond 2.7 weeks, which considered all the independent variables investigated. This underscores the critical importance of conducting disaggregated racial subgroup analysis in lung cancer treatment, rather than relying solely on aggregated general population data. Additionally, combining racial and general population findings for health policies and interventions may obscure the differential subgroup and racial differences necessary for effective and efficient policy decision-making. For instance, Holmes & Cohen used a nationally representative sample from the National Cancer Database, 2008–2013, and found that the median time to treatment initiation for non-small cell lung cancer was 8.2 days longer for African American patients compared to White patients [36]. Similarly, Cushman et al. (2021), which utilized the same dataset from 2004 to 2013, found that the median time to treatment initiation for African Americans and Hispanics was longer compared to other racial group [37]. Braithwaite et al. (2009) reported that Black communities in the U.S. faced more inadequate health care, low health education, and a shorter life expectancy for lung cancer than their White counterparts [38]. Addressing these disparities in Black communities could help reduce the risk of delayed treatment for invasive lung cancer. Additionally, the differences between aggregated population and subgroup analyses highlight the importance of examining disaggregated data to inform, targeted policy interventions, particularly with racial considerations. While general population analysis provides an overall view of lung cancer treatment initiation times, subgroup analysis reveals race-specific perspectives and disparities, enabling more tailored and effective policy interventions. Consistent with our findings, Roshini et al. (2022) highlighted the importance of disaggregating data to identify potential disparities in risk and protective factors, which can lead to better-informed, targeted interventions [39]. Our results further reinforce the need for additional research into subgroup disaggregation in population-based public health studies.

Married invasive lung cancer patients were 13% more likely to start treatment after 2.7 weeks compared to single or never-married patients in the general population. In the stratified racial subgroup analyses, married White patients had a 13% increased risk of delayed treatment initiation beyond 2.7 weeks, while married Black patients had a 16% increased risk compared to their single or never-married counterparts. Notably, the risk increase for married White patients was consistent with that observed in the general population. However, Black patients experienced a 3% higher risk of delayed treatment initiation compared to the general population. In contrast, Chen et al. (2021) found that marriage was associated with improved cancer-specific survival among patients who received early diagnosis and treatment with surgery [40]. This underscores the importance of early treatment for married individuals with invasive lung cancer. Although this study did not investigate the impact of financial stress or marital burden on treatment initiation, it is plausible that financial factors may contribute to treatment delays in Tennessee. Future research should explore this potential effect. Additionally, providing financial assistance to married patients could help reduce delays in treatment initiation and improve survival outcomes.

The county of residence (Appalachian or non-Appalachian) of invasive lung cancer patients in Tennessee had a significant influence on the time to treatment initiation. There was a statistically significant variation in the time to treatment initiation between the Appalachian and non-Appalachian counties in the general population sample and among White patients, but not among Black patients. Although no significant association was found between time to treatment initiation and the type of county of residence in Tennessee, patients in the Appalachian counties were 2% more likely to delay treatment or begin treatment after 2.7 weeks than those in non-Appalachian counties. Additionally, in the Appalachian county, White patients had a lower increased risk of late treatment compared to Black patients (2% vs. 5%). Research has shown that the Appalachian region of Tennessee experiences significant healthcare disparities, with the highest incidence of cancer and cancer mortality rates [41]. The variance in time to treatment between the two counties could be related to a lack of adequate cancer care resources, as observed in other Appalachian regions [42, 43]. A Study by Atkins et al. (2017)) revealed that lung cancer mortality increased with rurality, with rural patients diagnosed with non-small cell lung cancer undergoing fewer surgeries, leading to shorter median survival compared to urban patients [44]. These findings highlight the influence of place, particularly in rural communities, on lung cancer treatment. There is a need for tailored educational and early detection programs targeting at-risk populations to ensure equitable access to cancer care resources, as factors such as distance to care centers [45] may impact treatment timing. In addition, interventions such as local community resources and telemedicine have yielded success in improving rural cancer care [46–48], and could be adopted in Tennessee. Furthermore, streamlining care coordination efforts may aid in addressing the timeliness of treatment for patients, especially in rural and minority communities who may have difficulties navigating the health systems and care processes.

Health insurance status or coverage was associated with the time to treatment initiation of lung cancer. Patients with private insurance were 8% more likely to delay treatment for invasive lung cancer beyond 2.7 weeks than self-pay/uninsured patients in the general sample. This trend was also observed among White and Black patient subgroups, although not statistically significant. Conversely, patients with public insurance had a 6% decreased risk of delaying treatment compared to those with self-pay/uninsured status. This suggests that individuals with private insurance are more likely to delay treatment, possibly due to the cost of deductibles associated with private insurance [49, 50]. Future studies should investigate this further, particularly assessing out-of-pocket costs and streamline approval processes for tests and procedures necessary for cancer treatment.

Patients diagnosed with localized and regional stages of invasive lung cancer experienced a decreased risk of delaying treatment after 2.7 weeks (i.e., 34% and 18%, respectively) compared with those diagnosed at the distant stage. This finding may reflect the clinical urgency associated with distant stage or metastatic cancer, as observed in a systematic review by Hall et al. (2021) [51]. While White patients with localized stage invasive lung cancer had a decreased likelihood of delaying treatment compared to their Black counterparts (35% vs. 32%), Black patients had a decreased risk of late treatment for regional stage invasive lung cancer compared to White patients (17% vs. 22%). These disparities are concerning given that approximately 75% of lung cancers are diagnosed at the advanced or distant stage with a poor survival rate [9]. Despite significant improvements have been made in the oncological management of distant-stage lung cancer in recent years, more efforts are needed to facilitate early treatment.

### Limitations

This study is not without limitations. The data used is cross-sectional data, which presents some weaknesses in terms of making strong and accurate conclusions or decisions. Nonetheless, this research outlines tremendous findings about the time to treatment initiation of invasive lung cancer in Tennessee and provides the need for prospective cohort studies to enhance further understanding. We were also limited by some administrative variables such as SES data (e.g., individual-level educational attainment, income), healthcare access, and other lung cancer treatments received by patients beside surgery. Additionally, the data did not specify the type of lung cancer patients were diagnosed, which can be either small cell lung cancer or non-small cell lung cancer, each requiring different treatment modalities. Also, delay from diagnosis to treatment of lung cancer may occur in different ways, including delays in the first appointment of with a general practitioner, referral delays, and delays from referral to the first visit of with a specialist, as well as delays in treatment initiation. Unfortunately, we lacked specific data on the types of delays encountered by patients. Examination of specific types of treatment delay in future studies can help design a more specific tailored intervention to reduce the disparities in invasive lung cancer treatment initiation in Tennessee. Importantly, our study examined the time from diagnosis to definitive treatment of invasive lung cancer, focusing on periods beyond the median time of 2.7 weeks. Previous studies have reported this timeframe as a treatment delay for lung cancer [10–12]. However, this study did not examine the clinical implications of not receiving treatment within the median time of 2.7 weeks after diagnosis. Further, there are conflicting findings regarding whether the 2.7 weeks treatment delay time is detrimental to patient survival [12–14]. Despite these limitations, our findings emphasize the need for further research to investigate whether a 2.7 weeks delay in treatment initiation negatively affects the survival of invasive lung cancer patients in Tennessee.

## Conclusions

The varied factors impacting diagnosis and the complexities associated with lung cancer treatment underscore the need to understand how time to treatment affects survival outcomes, especially given the diversity in care resources across Appalachian counties in Tennessee. Invasive lung cancer patients in Tennessee who experienced delays in treatment initiation were more likely to be Blacks, <45 years old, married, and have private insurance. The findings from this study can aid clinicians and care coordination teams in identifying high-risk populations and developing comprehensive, tailored care plans based on patient demographics and rural-urban residency. Finally, this study highlights the need for improved cancer care resources in Black communities in Tennessee to ensure timely treatment of invasive lung cancer and equity of care for all cancer patients.

## Supporting information

S1 Methods Assessment of Cox proportional hazard assumptions of time to treatment initiation of invasive lung cancer, 2005–2015. (DOCX)

S1 Table Descriptives and bivariate non-parametric Kruskal-Wallis test analysis of time to treatment initiation of invasive lung cancer; *N* = 42,970. (DOCX)

S2 Table Multivariable Cox-proportional hazard model of time to treatment initiation of invasive lung cancer beyond 4.8 interquartile range weeks in the overall patient sample. *N* = 42,970.
(DOCX)

S3 Table Multivariable Cox-proportional hazard model assessment of time to treatment initiation of invasive lung cancer beyond interquartile range among White (4.7 weeks) and Black (5.5 weeks) patients in Tennessee. (DOCX)
