# Supplementary material for: Disparities in time to treatment initiation of invasive lung cancer among Black and White patients in Tennessee
Source: PLoS One. 2025 Jan 3;20(1):e0311186. doi: 10.1371/journal.pone.0311186 (PMC11698444; doi:10.1371/journal.pone.0311186)

**S1 Methods.** Assessment of Cox proportional hazard assumptions of time to treatment initiation of invasive lung cancer, 2005-2015

**Assumption 1. White Patients Data Time to Initiation Analysis Results**

> lccoxw <- coxph(Surv(WeekTrtDelay, Event) ~ ., data = coxlcw);lccoxw

Call:

coxph(formula = Surv(WeekTrtDelay, Event) ~ ., data = coxlcw)

coef exp(coef) se(coef) z p

Sex_CatF 0.005667 1.005683 0.015009 0.378 0.7057

Age_CatA1 0.100430 1.105646 0.060906 1.649 0.0992

Age_CatA2 0.034720 1.035329 0.030200 1.150 0.2503

Age_CatA3 -0.004084 0.995924 0.022847 -0.179 0.8581

Age_CatA4 0.045753 1.046816 0.018567 2.464 0.0137

Marital_CatD 0.033182 1.033739 0.029995 1.106 0.2686

Marital_CatM 0.118900 1.126257 0.025455 4.671 3e-06

Marital_CatW 0.016004 1.016133 0.030319 0.528 0.5976

Insure_CatPriv 0.060231 1.062082 0.040350 1.493 0.1355

Insure_CatPub -0.078420 0.924576 0.040594 -1.932 0.0534

County_CatApp 0.018160 1.018326 0.014548 1.248 0.2119

Stage_CatLoc -0.423737 0.654596 0.019445 -21.791 <2e-16

Stage_CatReg -0.192415 0.824964 0.018003 -10.688 <2e-16

RxSumSurgPSite_CatYes -0.010701 0.989356 0.017162 -0.624 0.5329

Likelihood ratio test=754.5 on 14 df, p=< 2.2e-16

n= 38490, number of events= 19749

> summary(lccoxw)

Call:

coxph(formula = Surv(WeekTrtDelay, Event) ~ ., data = coxlcw)

n= 38490, number of events= 19749

coef exp(coef) se(coef) z Pr(>|z|)

Sex_CatF 0.005667 1.005683 0.015009 0.378 0.7057

Age_CatA1 0.100430 1.105646 0.060906 1.649 0.0992 .

Age_CatA2 0.034720 1.035329 0.030200 1.150 0.2503

Age_CatA3 -0.004084 0.995924 0.022847 -0.179 0.8581

Age_CatA4 0.045753 1.046816 0.018567 2.464 0.0137 *

Marital_CatD 0.033182 1.033739 0.029995 1.106 0.2686

Marital_CatM 0.118900 1.126257 0.025455 4.671 3e-06 ***

Marital_CatW 0.016004 1.016133 0.030319 0.528 0.5976

Insure_CatPriv 0.060231 1.062082 0.040350 1.493 0.1355

Insure_CatPub -0.078420 0.924576 0.040594 -1.932 0.0534 .

County_CatApp 0.018160 1.018326 0.014548 1.248 0.2119

Stage_CatLoc -0.423737 0.654596 0.019445 -21.791 <2e-16 ***

Stage_CatReg -0.192415 0.824964 0.018003 -10.688 <2e-16 ***

RxSumSurgPSite_CatYes -0.010701 0.989356 0.017162 -0.624 0.5329

---

Signif. codes: 0 ‘***’ 0.001 ‘**’ 0.01 ‘*’ 0.05 ‘.’ 0.1 ‘ ’ 1

exp(coef) exp(-coef) lower .95 upper .95

Sex_CatF 1.0057 0.9943 0.9765 1.0357

Age_CatA1 1.1056 0.9044 0.9812 1.2458

Age_CatA2 1.0353 0.9659 0.9758 1.0985

Age_CatA3 0.9959 1.0041 0.9523 1.0415

Age_CatA4 1.0468 0.9553 1.0094 1.0856

Marital_CatD 1.0337 0.9674 0.9747 1.0963

Marital_CatM 1.1263 0.8879 1.0714 1.1839

Marital_CatW 1.0161 0.9841 0.9575 1.0783

Insure_CatPriv 1.0621 0.9415 0.9813 1.1495

Insure_CatPub 0.9246 1.0816 0.8539 1.0011

County_CatApp 1.0183 0.9820 0.9897 1.0478

Stage_CatLoc 0.6546 1.5277 0.6301 0.6800

Stage_CatReg 0.8250 1.2122 0.7964 0.8546

RxSumSurgPSite_CatYes 0.9894 1.0108 0.9566 1.0232

Concordance= 0.576 (se = 0.002 )

Likelihood ratio test= 754.5 on 14 df, p=<2e-16

Wald test = 745.8 on 14 df, p=<2e-16

Score (logrank) test = 753.3 on 14 df, p=<2e-16

> ggforest(lccoxw, data = coxlc)

> ggforest(lccoxw, data = coxlcw)

> test.ph <- cox.zph(lccoxw) #testing Cox-PH assumption

> test.ph

chisq df p

Sex_Cat 9.78 1 0.05187

Age_Cat 8.34 4 0.07989

Marital_Cat 7.52 3 0.05705

Insure_Cat 5.20 2 0.07427

County_Cat 2.47 1 0.11598

Stage_Cat 4.22 2 0.12124

RxSumSurgPSite_Cat 2.09 1 0.14827

GLOBAL 11.00 14 0.68603

> ggcoxzph(test.ph)

| **Independent variables** | **Chi-square value** | **Degree of freedom** | **p-value** |
| --- | --- | --- | --- |
| Sex | 2.57 | 1 | 0.051 |
| Age at diagnosis | 2.95 | 4 | 0.079 |
| Marital Status | 2.65 | 3 | 0.057 |
| Insurance Coverage | 3.47 | 2 | 0.074 |
| County of residence | 1.08 | 1 | 0.115 |
| Stage of Cancer | 2.08 | 2 | 0.121 |
| Surgical Treatment | 3.06 | 1 | 0.148 |
| GLOBAL Test | 8.71 | 14 | 0.686 |


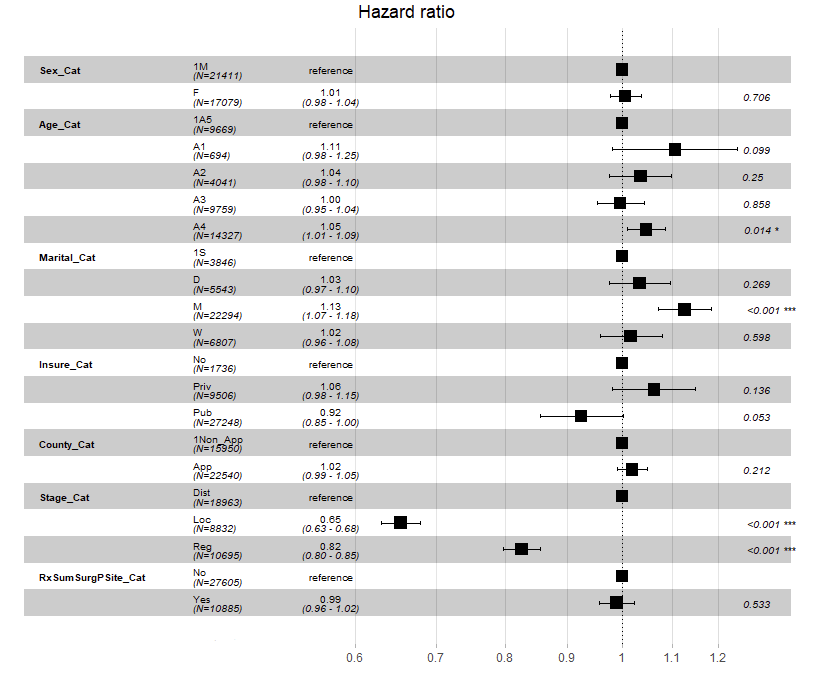


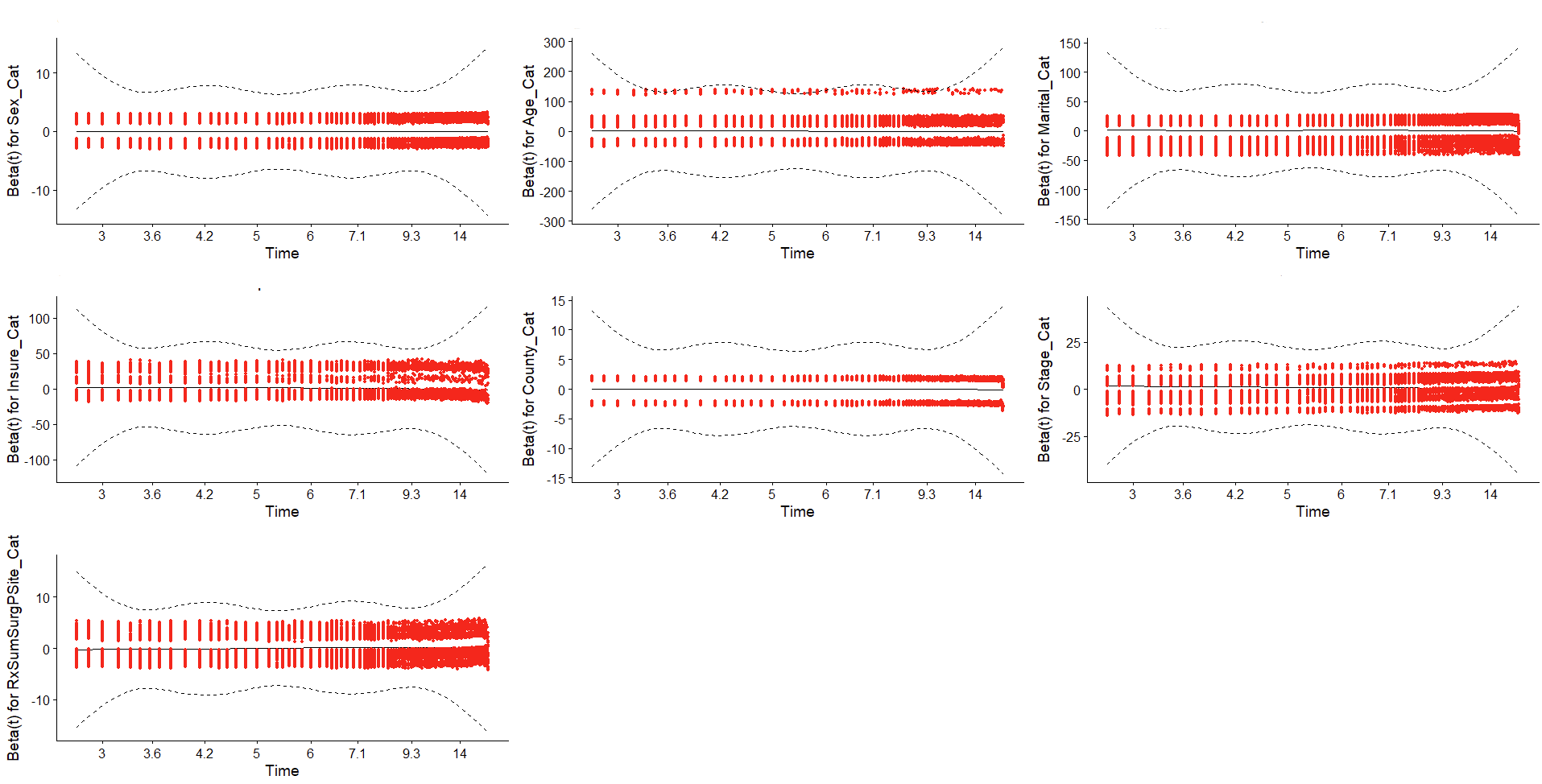


**Assumption 2. Black Patients Data Time to Treatment Initiation Analysis Results**

> lccox <- coxph(Surv(WeekTrtDelay, Event) ~ ., data = coxlc);lccox

Call:

coxph(formula = Surv(WeekTrtDelay, Event) ~ ., data = coxlc)

coef exp(coef) se(coef) z p

Sex_CatF -0.04214 0.95873 0.04422 -0.953 0.34056

Age_CatA1 0.33694 1.40065 0.16806 2.005 0.04497

Age_CatA2 -0.13594 0.87290 0.08489 -1.601 0.10930

Age_CatA3 -0.02585 0.97448 0.07251 -0.357 0.72144

Age_CatA4 -0.03494 0.96566 0.06543 -0.534 0.59329

Marital_CatD 0.04083 1.04167 0.06501 0.628 0.53000

Marital_CatM 0.14804 1.15956 0.05313 2.786 0.00533

Marital_CatW 0.15276 1.16504 0.06954 2.197 0.02804

Insure_CatPriv 0.15072 1.16268 0.08495 1.774 0.07602

Insure_CatPub -0.02669 0.97366 0.08404 -0.318 0.75080

County_CatApp 0.05093 1.05225 0.05281 0.964 0.33481

Stage_CatLoc -0.39143 0.67609 0.06223 -6.290 3.16e-10

Stage_CatReg -0.25392 0.77576 0.05268 -4.820 1.43e-06

RxSumSurgPSite_CatYes -0.07422 0.92846 0.05333 -1.392 0.16401

Likelihood ratio test=102.7 on 14 df, p=1.463e-15

n= 4480, number of events= 2308

> ggforest(lccox, data = coxlc)

> test.ph <- cox.zph(lccox) #testing Cox-PH assumption

> test.ph

chisq df p

Sex_Cat 2.57 1 0.10857

Age_Cat 2.95 4 0.56605

Marital_Cat 2.65 3 0.44855

Insure_Cat 3.47 2 0.17640

County_Cat 1.08 1 0.29897

Stage_Cat 2.08 2 0.35323

RxSumSurgPSite_Cat 3.06 1 0.08032

GLOBAL 8.71 14 0.84944

> ggcoxzph(test.ph)#cox assumption

| **Independent variables** | **Chi-square value** | **Degree of freedom** | **p-value** |
| --- | --- | --- | --- |
| Sex | 2.57 | 1 | 0.109 |
| Age at diagnosis | 2.95 | 4 | 0.566 |
| Marital Status | 2.65 | 3 | 0.449 |
| Insurance Coverage | 3.47 | 2 | 0.176 |
| County of residence | 1.08 | 1 | 0.299 |
| Stage of Cancer | 2.08 | 2 | 0.353 |
| Surgical Treatment | 3.06 | 1 | 0.080 |
| GLOBAL Test | 8.71 | 14 | 0.849 |


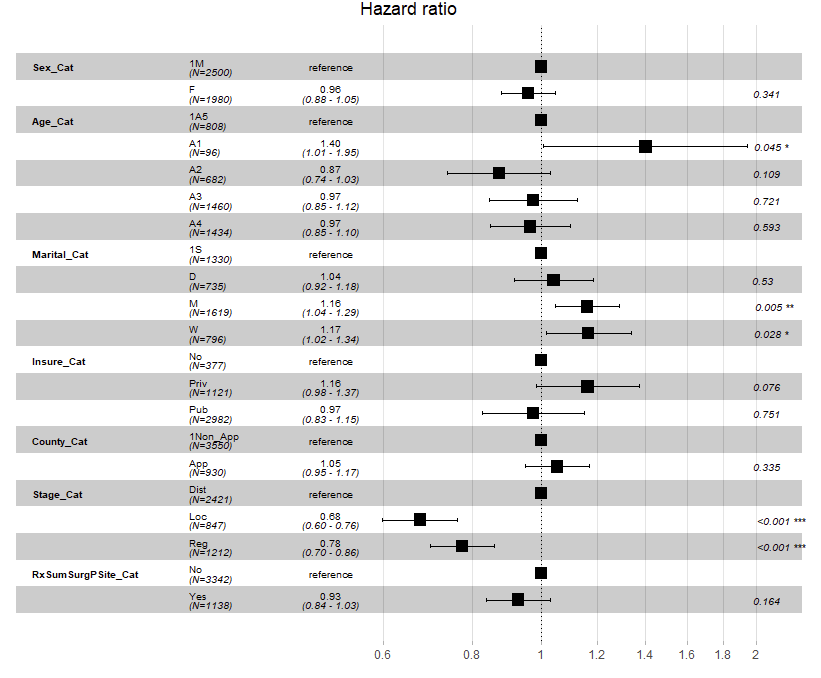


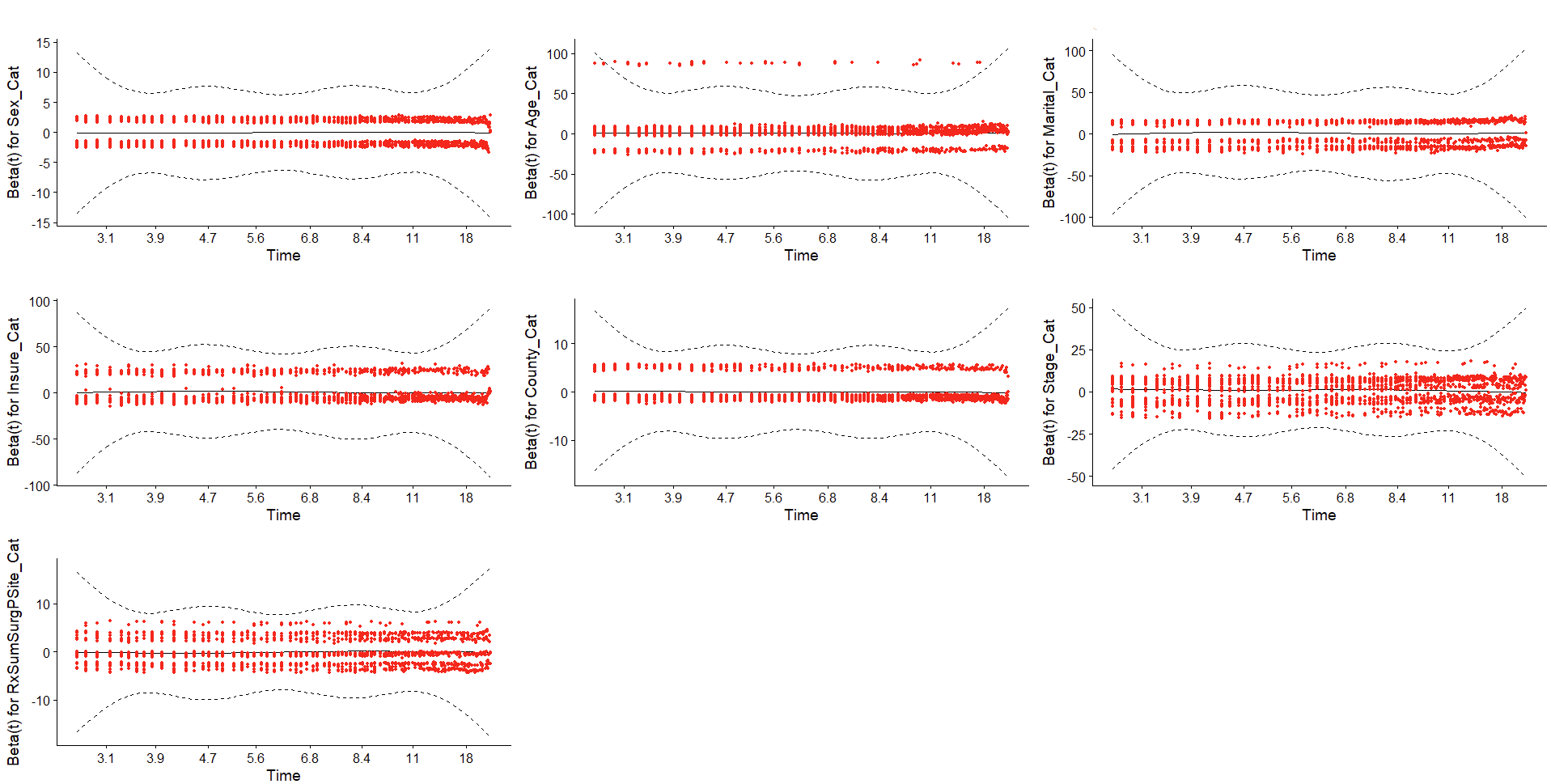

Supplement: S1 Methods — (DOCX) [file pone.0311186.s001.docx]
