# Supplementary material for: Disparities in time to treatment initiation of invasive lung cancer among Black and White patients in Tennessee
Source: PLoS One. 2025 Jan 3;20(1):e0311186. doi: 10.1371/journal.pone.0311186 (PMC11698444; doi:10.1371/journal.pone.0311186)
Supplement: S1 Table — (DOCX) [file pone.0311186.s002.docx]

| **S2 Table.** Descriptives and bivariate non-parametric Kruskal-Wallis test analysis of time to treatment initiation of invasive lung cancer. *N*=42,970 | | | | | | |
| --- | --- | --- | --- | --- | --- | --- |
| **Characteristics** | **Total Patient Sample *N*=42970)** | | **White Patient (*N*=38490)** | | **Black Patients (*N*=4480)** | |
| **Independent Variables** | **n (%)** | **P-value** | **n (%)** | **P-value** | **n (%)** | **P-value** |
| **Sex** |  | 0.563 |  | 0.472 |  | **<0.001** |
| Male | 24911 (55.6) |  | 21411 (55.6) |  | 2500 (55.8) |  |
| Female | 19059 (44.4) |  | 17079 (44.4) |  | 1980 (44.2) |  |
| **Age at Diagnosis** |  | **<0.001** |  | **<0.001** |  | **<0.001** |
| <45 | 790 (1.8) |  | 694 (1.8) |  | 96 (2.1) |  |
| 45-54 | 4723 (11.0) |  | 4041 (10.5) |  | 682 (15.2) |  |
| 55-64 | 11219 (26.1) |  | 9759 (25.4) |  | 1460 (32.0) |  |
| 65-74 | 15761 (36.7) |  | 14327 (37.2) |  | 1434 (32.0) |  |
| ≥75 | 10477 (24.4) |  | 9669 (25.1) |  | 808 (18.0) |  |
| **Race** |  | **0.003** |  |  |  |  |
| White | 38490 (89.6) |  | - |  | - |  |
| Black | 4480 (10.4) |  | - |  | - |  |
| **Marital Status** |  | 0.182 |  | **0.009** |  | **0.028** |
| Single/Never Married | 5276 (12.0) |  | 3846 (10.0) |  | 1330 (29.7) |  |
| Married/Common Law | 23913 (55.7) |  | 22294 (57.9) |  | 1619 (36.1) |  |
| Divorced/Separated | 6278 (14.6) |  | 5543 (14.4) |  | 735 (16.4) |  |
| Widowed | 7603 (17.7) |  | 6807 (17.7) |  | 796 (17.8) |  |
| **County of Residence** |  | **0.005** |  | **<0.001** |  | 0.398 |
| Appalachian | 23470 (54.6) |  | 22540 (58.6) |  | 930 (20.8) |  |
| non-Appalachian | 19500 (45.4) |  | 15,950 (41.4) |  | 3550 (79.2) |  |
| **Health Insurance Type** |  | **<0.001** |  | **<0.001** |  | 0.294 |
| Self-Pay/Uninsured | 2113 (4.9) |  | 1736 (4.5) |  | 377 (8.4) |  |
| Public | 30230 (70.4) |  | 27248 (70.8) |  | 2982 (66.6) |  |
| Private | 10627 (24.7) |  | 9506 (24.7) |  | 1121 (25.0) |  |
| **Cancer Stage** |  | **<0.001** |  | **<0.001** |  | **<0.001** |
| Localized | 9679 (22.5) |  | 8832 (22.9) |  | 847 (18.9) |  |
| Regional | 11907 (27.7) |  | 10695 (27.8) |  | 1212 (24.1) |  |
| Distant | 21384 (49.8) |  | 18963 (49.3) |  | 2421 (54.0) |  |
| **Surgical Treatment** |  | 0.085 |  | 0.150 |  | 0.217 |
| Yes | 12023 (28.0) |  | 10885 (28.3) |  | 1138 (25.4) |  |
| No | 30947 (72.0) |  | 27605 (71.7) |  | 3342 (74.6) |  |
| **Time to Treatment Initiation** |  | - |  | - |  | - |
| ≤2.7 weeks | 20913 (48.7) |  | 18741 (48.7) |  | 2172 (48.5) |  |
| >2.7 weeks | 22057 (51.3) |  | 19749 (51.3) |  | 2308 (51.5) |  |
| Statistical analysis performed= Kruskal-Wallis Tests  Public insurance= (Indian Health Service, Medicaid, Medicare, Veterans’ Affairs)  Private insurance= (Fee for Service, HMO, Managed Care, PPO)  Bold= Statistical significance, p <0.05. | | | | | | |
