# Supplementary material for: Disparities in time to treatment initiation of invasive lung cancer among Black and White patients in Tennessee
Source: PLoS One. 2025 Jan 3;20(1):e0311186. doi: 10.1371/journal.pone.0311186 (PMC11698444; doi:10.1371/journal.pone.0311186)
Supplement: S2 Table — N = 42,970. (DOCX) [file pone.0311186.s003.docx]

**S3 Table.** Multivariable Cox-proportional hazard model of time to treatment initiation of invasive lung cancer beyond 4.8 interquartile range weeks in the overall patient sample. *N*= 42,970

| **Independent Description** | **aHR (95% CI)** | **P-value** |
| --- | --- | --- |
| **Sex** |  |  |
| Male | Ref | - |
| Female | 1.02 (0.99, 1.06) | 0.204 |
| **Age at Diagnosis** |  | <0.173 |
| <45 | 1.04 (0.89, 1.21) | 0.624 |
| 45-54 | 0.96 (0.89, 1.03) | 0.210 |
| 55-64 | 0.97 (0.92, 1.03) | 0.344 |
| 65-74 | 1.02 (0.98, 1.07) | 0.314 |
| ≥75 | Ref | - |
| **Race** |  | **<0.001** |
| White | Ref | - |
| Black | 0.83 (0.79, 0.88) | **<0.001** |
| **Marital status** |  | **<0.001** |
| Single/Never Married | Ref | - |
| Married/Common Law | 1.12 (1.06, 1.19) | **<0.001** |
| Divorced/Separated | 1.04 (0.98, 1.12) | 0.213 |
| Widowed | 1.03 (0.97, 1.05) | 0.355 |
| **County of Residence** |  |  |
| Appalachian | 1.01 (0.98, 1.05) | 0.568 |
| non-Appalachian | Ref | - |
| **Health Insurance Type** |  | **<0.001** |
| Self-Pay/Uninsured | Ref | - |
| Public | 0.94 (0.84, 1.01) | 0.083 |
| Private | 1.01 (0.92, 1.10) | 0.907 |
| **Cancer Stage** |  | **<0.001** |
| Localized | 0.75 (0.71, 0.78) | **<0.001** |
| Regional | 0.91 (0.87, 0.95) | **<0.001** |
| Distant | Ref | - |
| **Surgical Treatment** |  |  |
| Yes | 1.07 (1.03, 1.12) | **<0.001** |
| No | Ref | - |

Statistical analysis performed= Multivariable Cox proportional hazard regression analysis.

Ref= Reference group; aHR= Adjusted Hazard ratio; CI= Confidence interval.

Public insurance= (Indian Health Service, Medicaid, Medicare, Veterans’ Affairs)

Private insurance = (Fee for Services, HMO, Managed Care, PPO).

Bold= Statistical significance, p <0.05.
