# Supplementary material for: Disparities in time to treatment initiation of invasive lung cancer among Black and White patients in Tennessee
Source: PLoS One. 2025 Jan 3;20(1):e0311186. doi: 10.1371/journal.pone.0311186 (PMC11698444; doi:10.1371/journal.pone.0311186)
Supplement: S3 Table — (DOCX) [file pone.0311186.s004.docx]

**S4 Table.** Multivariable Cox-proportional hazard model assessment of time to treatment initiation of invasive lung cancer beyond interquartile range among White (4.7 weeks) and Black (5.5 weeks) patients in Tennessee.

|  | **White Patient Model (*n* = 38,490)** | | **Black Patient Model *(n* = 4,480)** | |
| --- | --- | --- | --- | --- |
| **Variable Description** | **aHR (95% CI)** | **P-value** | **aHR (95% CI)** | **P-value** |
| **Sex** |  |  |  |  |
| Male | Ref | - | Ref | - |
| Female | 1.03 (0.99, 1.07) | 0.177 | 0.97 (0.87, 1.09) | 0.628 |
| **Age at Diagnosis** |  | 0.144 |  | 0.259 |
| <45 | 1.11 (0.88, 1.23) | 0.602 | 1.07 (0.64, 1.78) | 0.793 |
| 45-54 | 1.04 (0.91, 1.06) | 0.691 | 0.79 (0.63, 0.98) | **0.035** |
| 55-64 | 1.00 (0.93, 1.04) | 0.508 | 0.86 (0.71, 1.04) | 0.125 |
| 65-74 | 1.05 (0.99, 1.09) | 0.092 | 0.90 (0.76, 1.07) | 0.230 |
| ≥75 | Ref | - | Ref | - |
| **Marital Status** |  | **<0.001** |  | 0.611 |
| Single/Never Married | Ref | - | Ref | - |
| Married/Common Law | 1.13 (1.06, 1.2) | **<0.001** | 1.08 (0.93, 1.22) | 0.359 |
| Divorced/Separated | 1.06 (0.98, 1.14) | 0.151 | 0.99 (0.84, 1.16) | 0.858 |
| Widowed | 1.03 (0.95, 1.10) | 0.525 | 1.09 (0.91, 1.30) | 0.258 |
| **County of Residence** |  |  |  |  |
| Appalachian | 1.02 (0.98, 1.05) | 0.390 | 1.02 (0.88, 1.17) | 0.831 |
| non-Appalachian | Ref | - | Ref | - |
| **Health Insurance Type** |  | **0.005** |  | **0.053** |
| Self-Pay/Uninsured | Ref | - | Ref | - |
| Public | 0.93 (0.84, 1.03) | 0.181 | 0.86 (0.69, 1.06) | 0.149 |
| Private | 1.01 (0.91, 1.12) | 0.858 | 1.02 (0.82, 1.27) | 0.827 |
| **Cancer Stage** |  | **<0.001** |  | **<0.001** |
| Localized | 0.75 (0.71, 0.78) | **<0.001** | 0.75 (0.65, 0.88) | **<0.001** |
| Regional | 0.91 (0.88, 0.96) | **<0.001** | 0.80 (0.70, 0.92) | **0.002** |
| Distant | Ref | - | Ref | - |
| **Surgical Treatment** |  |  |  |  |
| Yes | 1.08 (1.04, 1.12) | **<0.001** | 1.03 (0.90, 1.17) | 0.656 |
| No | Ref | - | Ref | - |
| Statistical analysis performed= Multivariable Cox proportional hazard regression analysis  Ref= Reference group; aHR= Adjusted Hazard ratio; CI= Confidence interval  Public insurance= (Indian Health Service, Medicaid, Medicare, Veterans’ Affairs)  Private insurance= (Fee for Service, HMO, Managed Care, PPO)  Bold= Statistical significance, p <0.05. | | | | |
